# MED22/401: Hypertext Atlas For Pathology Education

**DOI:** 10.2196/jmir.1.suppl1.e64

**Published:** 1999-09-19

**Authors:** J Feit, L Matyska

**Affiliations:** ^1^Masaryk UniversityBrnoCzech Republic

**Keywords:** Pathology, Telepathology, Dermatopathology, Educational Technology, Multimedia

## Abstract

**Introduction:**

Macro- and microscopic pictures play the key role in the pathology diagnosis and education. Collections of annotated digital histological images are being prepared and an interface offering convenient way of dealing with high resolution images and their descriptions was developed. The atlas will be primary used in pre-graduate course of pathology at the University.

**Methods:**

Leica DMLB microscope equipped with the Leica~S1 scanning camera is used to obtain pictures at the resolution of up to5112*5112 pixels, 3*12~bit color. These pictures are digitally manipulated (esp. contrast enhancement and sharpening), downsized to 3000*3000 pixels, 3*8 bit color and archived. Subsequently, these raw images are compressed using JPEG (JFIF lossy format) to 900*900 and 2000*2000 pixel images used in the actual collection. These pictures are logically glued together and described in the atlas source text prepared using the TeX/LaTeX typesetting system. LaTeX2HTML converter is used to produce the actual hypermedia document, while the same source can be used to obtain a more classical printed version of the atlas. The static character of the HTML hypertext document is made dynamic through JavaScript functions, which add the required interactivity.

**Results:**

The atlas is accessible using standard JavaScript enabled Web browsers. There is always one main window with the text, table of content, index, active section headers and other hypertext links, and links to individual images. All the images are displayed in separate windows. The 900*900 pixels images are used as standard resolution pictures, the higher resolution images (2000*2000) are displayed on demand in a separated ``magnifying glass'' window. This interface can be used to compare several pictures displaying them in the same time and providing also a tool to focus on the most important parts of the large windows. Use of the higher resolution images leads to no information loss through this process. The interface could mimic the fine focusing of the microscope that provided the picture and is a series of images taken in consecutive planes of focus.

**Discussion:**

The atlas was primary produced as a stand-alone package, stored on local disc or CD-ROM. In this form it is suitable for pre-graduate and initial postgraduate education. Currently, it is being converted into very flexible, easily available and expandable diagnostic tool available through Internet. The Internet accessible atlas may be easily updated and expanded with new images and text. Images at different compression levels could be stored, the low resolution one used to fast scan and the highest resolution images used for detailed comparison with unknown specimen. Through the very high speed network (e.g. TEN-155) it will be possible to transfer even the raw data images with resolution above3000*3000 pixels.

